# Impact of serum luteinizing hormone level and increment on the day of endometrial transformation on frozen–thawed single blastocyst transfer outcome in the hormone replacement cycle

**DOI:** 10.1186/s13048-025-01905-x

**Published:** 2025-11-27

**Authors:** Kang Yu, Junhong Zhou, Jing Ju, Ailing Han

**Affiliations:** 1 Center of Reproductive Medicine, Weifang People’s Hospital, Weifang, 261041 China; 2 Department of Reproductive Medicine, Sunshine Union Hospital, Weifang, 261000 China; 3 Outpatient department, Weifang People’s Hospital, Weifang, 261041 China

**Keywords:** Clinical pregnancy rate, Frozen-thawed embryo transfer, Hormone replacement cycle, Luteinizing hormone

## Abstract

**Objective:**

To analyze the effect of serum luteinizing hormone (LH) level and increment on the day of endometrial transformation on the outcome of frozen-thawed embryo transfer during the hormone replacement cycle.

**Method:**

We conducted a retrospective cohort study to analyze the clinical data of 845 women who underwent frozen-thawed embryo transfer during the hormone replacement cycle at Weifang People’s Hospital from January 2019 to December 2021. The women with a median serum LH level 11.97 U/L (0.96 U/L − 24.17 U/L) on the day of endometrial transformation were enrolled. The serum LH levels on the day of endometrial transformation were divided into two groups according to the median. If there was a difference in pregnancy outcome between the high and low LH levels, the range of LH levels was further narrowed. The range of LH between 0 and 25 U/L was divided into one group according to every 5 U/L, and the LH levels of all patients were divided into five groups according to the low to high LH levels. The correlation between the outcome of frozen-thawed embryo transfer and LH level was compared.

**Results:**

Women with serum LH > 11.97 U/L on the day of endometrial transformation had higher human chorionic gonadotropin (HCG) positive rates (*P* = 0.012) and clinical pregnancy rates (*P* = 0.022) than those with LH ≤ 11.97 U/L. The LH > 15 U/L group had a higher HCG positive rate (OR = 1.274, 95% CI = 1.034–1.699, *P* = 0.044), while the LH > 20 U/L group had a higher clinical pregnancy rate (OR = 1.879, 95% CI = 1.124–3.287, *P* = 0.019).

**Conclusions:**

Higher serum LH levels and increment on the day of endometrial transformation in the hormone replacement cycle are positively correlated with a higher clinical pregnancy rate in women with normal basal endocrine levels undergoing frozen-thawed embryo transfer. This suggests that elevating the LH levels prior to the transfer of frozen embryos may increase the likelihood of successful embryo implantation and aid in identifying the optimal implantation window.

**Trial registration:**

ChiCTR2200060489.

## Introduction

Frozen-thawed embryo transfer (FET) is an important component of in vitro fertilization-embryo transfer (IVF-ET) technology. Using a hormone replacement cycle allows for more flexible transplantation scheduling and reduces monitoring time. Hormone replacement programs were first proposed by some researchers in the 1980 s to simulate hormone levels and endometrium changes in the normal menstrual cycle, with the aim to facilitate the successful implantation of frozen-thawed embryos [1]. Some researchers have conducted a related study in recent years on optimizing this program, to determine the appropriate hormone level and timing of endometrial transformation to improve the pregnancy outcome of FET in the hormone replacement cycle [2]. Luteinizing hormone (LH) is a gonadotropin secreted by the anterior pituitary gland under the stimulation of hypothalamic gonadotropin-releasing hormone. It plays a critical role in estrogen synthesis, oocyte maturation, fertilization, and embryo quality in females. In recent years, studies have revealed that LH exerts functions beyond gonadal tissues, influencing various non-reproductive organs. Notably, functional LH receptor (LH-R) gene expression has been identified in both the human myometrium and endometrium [3]. Upon binding to LH, the LH-R activates multiple signaling pathways, including protein kinase A, protein kinase C, cyclooxygenase-2 (COX-2), and MAP kinase cascades, which regulate prostaglandin synthesis [4]. This molecular mechanism contributes to establishing optimal conditions for embryo implantation and plays a significant role in early pregnancy maintenance. In this study, we explored the correlation between different serum luteinizing hormone (LH) levels on the day of endometrial transformation, the transplantation outcome of FET in the hormone replacement cycle, and the effect of LH increment on transplantation outcome.

## Data and date

### Study participants

We conducted a retrospective cohort study and selected 845 women who underwent FET in the hormone replacement cycle at the Reproductive Medicine Center of Weifang People’s Hospital from January 2019 to February 2022. Inclusion criteria: women aged 21*–*39 who underwent FET in the hormone replacement cycle, transferred one high-quality blastocyst, and had an endometrium ≥ 8 mm thick on the day of progesterone conversion. Exclusion criteria: (1) Comorbidities such as hypertension, diabetes, hyperthyroidism, hypothyroidism, hyperprolactinemia, polycystic ovary syndrome, hypogonadotropic hypogonadism, etc. (2) Untreated uterine malformation, giant uterine fibroids, endometrial tuberculosis, intrauterine adhesion, adenomyosis, and hydrosalpinx. (3) Chromosome abnormality in either spouse. (4) Women with a history of miscarriages and repeated implantation failure. This study was approved by the Ethics Committee of Reproductive Medicine of Weifang People’s Hospital, and all participants gave informed consent.

### Grouping method

To analyze the relationship between serum LH level and the frozen embryo transfer outcome, the participants were divided into two groups based on the median serum LH level on the day of endometrial transformation, which was defined as the first day of progesterone administration, and the embryo transfer outcomes were compared. The LH level range was narrowed based on the difference in pregnancy outcomes between different LH levels. The participants were divided into five groups based on the serum LH level on the day of endometrial transformation for every 5 U/L, within the range of 0–25 U/L. The participants in the five groups were sorted from low to high based on the LH level and compared to find a correlation between FET outcomes and LH levels.

Additionally, to analyze the change rule of serum LH level on FET in the hormone replacement cycle and to understand whether the increment of LH is related to the FET outcome, two representative time points were selected, viz., basal LH value (D0) and endometrial transformation day (D1). The participants were grouped following different pregnancy outcome variables into the biochemical pregnancy group, non-biochemical-pregnancy group, clinical pregnancy group, and non-clinical–pregnancy group.

### Treatment plan

During the FET cycle, the participants underwent blood tests on the second or third day of their menstrual cycle to measure the basal LH level, referred to as the basal value (D0 time). Starting from the 2nd − 3rd day of menstruation, all participants took Femoston (Estradiol Tablets/Estradiol and Dydrogesterone Tablets, Dutch Abbott, 2 mg/tablet) orally; the initial dose of Femoston red tablets (Estradiol Valerate Tablets, 2 mg/tablet) was 4 mg/d. After 10 days, the endometrial thickness was monitored using B-ultrasound, and the medication was increased to 6 mg/d based on the examination results. When the endometrial thickness was ≥ 8 mm and the medication days were ≥ 10, serum LH levels were measured on D1. At the same time, the participants began taking Femoston yellow tablets (Estradiol 2 mg/tablet and dydrogesterone 10 mg/tablet) and were administered progesterone injection 40 mg/d intramuscularly for endometrial transformation. Blood was collected for freezing and uniformly tested using the automated chemiluminescence method (Beckmann Corporation, USA). The serum LH levels were determined throughout the process using the same method. On the fifth day of transformation, FET was performed using ultrasound guidance, and a survived high-quality blastocyst from the fifth or sixth day of development was transplanted. Luteal-phase support after transplantation: Femoston yellow tablets 4 mg/d + progesterone injection 40 mg/d.

#### Detection of pregnancy and its outcome

Serum β-HCG levels were measured 14 days post-transplantation; β-HCG >10 U/L was considered as HCG positive. Serum β-HCG levels were higher than the normal range on the 14th day after transplantation, and then gradually decreased. Biochemical pregnancy was determined when ultrasonography did not detect a gestational sac echo; 28 days after transplantation, transvaginal ultrasound was performed to diagnose intrauterine or extrauterine gestational sac as clinical pregnancy. Luteal support was continued until the 10th week of pregnancy upon confirming intrauterine pregnancy, with gradual reduction and discontinuation of the medication. Termination of pregnancy within 12 weeks of gestation was considered early abortion [5]. 

The Gardner’s grading system was used for blastocyst scoring, which is divided into six grades based on the formation of the blastocyst cavity. Grade 3–6 blastocysts were then scored based on inner cell mass and trophectoderm cell quality [2]. Blastocysts that scored ≥ grade 4 (4BC), were defined as high-quality blastocysts. Each participant was transferred in this study with 1 D5 or D6 high-quality survived blastocyst blastocyst.

### Observation indexes

HCG positive rate, biochemical and clinical pregnancy rate, and early abortion rate. HCG positive rate: number of HCG positive cycles/transplantation cycles × 100%; Biochemical pregnancy rate: number of biochemical pregnancy cycles/transplantation cycles × 100%; Clinical pregnancy rate: number of clinical pregnancy cycles/transplantation cycles × 100%; Early abortion rate: number of early abortion cycles/clinical pregnancy cycles × 100%.

### Statistical methods

Statistical analyses were conducted using DMSAS software. Continuous normal distribution data are represented by mean ± standard deviation, while non-normal distribution measurement data are represented by median (25th percentile, 75th percentile) (M [Q1, Q3]). Normal distribution inter-group comparison was performed using the *t*-test, non-normal distribution data was compared using the Mann-Whitney U test. The count data are presented as rate (%), and the chi-squared test was used for comparison between groups. Logistic regression was used to adjust for the effects of age, body mass index (BMI), anti-Mullerian hormone (AMH), and basal LH levels. The OR value and 95% confidence intervals (CI) were calculated. The difference is statistically significant when *P* < 0.05

## Results

### Comparison of general data and embryo transfer outcomes in participants with different LH levels

There were 845 eligible frozen embryo transfer cycles. To analyze the relationship between LH level on the day of endometrial transformation and assisted pregnancy outcome, the participants were divided based on LH level into the LH ≤ 11.97 U/L group and LH > 11.97 U/L group based on the median LH level of 11.97 U/L, as shown in Table 1. There was no statistically significant difference in age, body mass index (BMI), basal endocrine (follicle-stimulating hormone (FSH), LH, estradiol (E2)), anti-mullerian hormone (AMH), endometrial thickness on the day of progesterone transformation, in vitro fertilization (IVF), and intracytoplasmic sperm injection (ICSI) between the two groups (all *P* > 0.05). Refer to Table 1 for details.

**Table 1:** Comparison of luteinizing hormone (LH) on the day of endometrial transformation and primary conditions of LH group with frozen embryo transfer outcome

Item	LH>11.97 U/L	LH ≤ 11.97 U/L	t/U χ2	*P* value
Number of cases	376	469		
Age	34.18 ± 5.08	34.97 ± 4.33	0.17	0.861
BMI	24.48 ± 3.54	22.77 ± 3.61	0.84	0.128
Basal FSH level	6.31 ± 1.84	6.49 ± 1.92	0.75	0.253
Basal LH level	4.87 ± 3.28	4.58 ± 3.57	0.62	0.325
Basal E2 level	139.33 ± 49.48	142.15 ± 36.91	0.48	0.527
AMH	3.14 ± 1.37	3.08 ± 1.43	0.65	0.296
Endometrial thickness on transformation day	0.95 ± 0.15	0.93 ± 0.17	0.35	0.628
IVF(%)	82.71(311/376)	79.96(375/469)	0.24	0.641
ICSI(%)	17.29(65/376)	20.04(94/469)	0.21	0.650
HCG positive rate (%)	67.29(253/376)	50.53(237/469)	6.27	0.012
Clinical pregnancy rate (%)	65.96(248/376)	48.19(226/469)	6.09	0.022
Early abortion rate (%)	11.29(28/248)	11.06(25/226)	0.19	0.645

Positive number/total number indicated in ()

### Effect of distribution of serum LH levels on the day of endometrial transformation on transplant outcome

The participants were divided into five groups based on the serum LH level on the day of endometrial transformation for every 5 U/L, within the range of 0–25 U/L, The participants in the five groups were sorted from low to high based on the LH level. Finally, there were 73 cases (8.64%) in group A (LH 0–4.9 U/L), 391 cases (46.27%) in group B (LH 5.0–9.9 U/L), 224 cases (26.51%) in group C (LH 10.0–14.9 U/L), 111 cases (13.14%) in group D (LH 15.0–19.9 U/L), and 46 cases (5.44%) in group E (LH 20.0–25 U/L). The general conditions of the study participants in the five groups were included in the logistic regression analysis; group B had the most participants, thus it was used as a reference. After preliminary exclusion, the age, basal LH level, BMI, and AMH were statistically significant in all the related variables in the regression model. However, basal FSH level, basal E2 level, and endometrial transformation thickness on the transformation day were not statistically significant. Therefore, only five variables—age, basal LH level, BMI, AMH, and LH level on the day of endometrial transformation were included in the three transplantation outcome-related indicators—HCG positive rate, clinical pregnancy rate, and early miscarriage rate. The adjusted OR value was obtained after adjusting for age, basal LH level, BMI, and AMH confounding factors. For HCG positive rate, OR (95% CI) in group A = 0.798 (0.603–0.847), in group D = 1.274 (1.034–1.699), and in group E = 1.702 (1.255–2.792), all of which were statistically significant (*P* < 0.05); For clinical pregnancy rate, OR (95% CI) in group A = 0.802 (0.688–0.979) and OR (95% CI) in group E = 1.903 (1.124–3.287), and the difference was statistically significant (*P* < 0.05). Refer to Table 2 for details.

**Table 2:** Relationship between LH level on the day of endometrial transformation and frozen embryo transfer outcome

Item	Group	Before correction			After correction		
		*P*	OR	95%CI	*P*	OR	95%CI
HCG positive rate	a	0.669	1.179	0.947 ~ 1.459	0.039	0.798	0.603 ~ 0.847
	b		1.000			1.000	
	c	0.658	1.241	0.973 ~ 1.578	0.557	0.991	0.819 ~ 1.205
	d	1.104	1.506	1.059 ~ 2.141	0.044	1.274	1.034 ~ 1.699
	e	0.037	2.012	1.165 ~ 3.459	0.038	1.702	1.255 ~ 2.792
Clinical pregnancy rate	a	0.546	1.157	0.927 ~ 1.456	0.045	0.802	0.688 ~ 0.979
	b		1.000			1.000	
	c	0.455	1.244	0.963 ~ 1.607	0.361	1.029	0.861 ~ 1.279
	d	0.377	1.335	0.951 ~ 1.890	0.351	1.140	0.827 ~ 1.571
	e	0.011	2.099	1.403 ~ 3.847	0.019	1.879	1.124 ~ 3.287

Group A: LH 0–4.9 U/L, Group B: LH 5.0–9.9 U/L, Group C: LH 10.0–14.9 U/L, Group D: LH 15.0–19.9 U/L, Group E: LH 20.0–24.9 µg/L

### Effect of LH increment on outcome in FET cycles

The participants were divided into the HCG positive group and HCG negative group, as well as the clinical pregnancy group and non-clinical pregnancy group, based on different outcome variables of the frozen embryo transfer. The changes in serum LH level at two time points were measured for each group (Table 3). When comparing the absolute difference in LH increment between the two time points (D1 compared with D0), it was found that the LH increment in the HCG positive group and clinical pregnancy group was significantly higher than that in the HCG negative group and non-clinical pregnancy group (D1 compared with D0 *P* < 0.05 in HCG positive group; D1 compared with D0 *P* < 0.01 in clinical pregnancy group).

**Table 3:** Effect of LH amplification on frozen embryo transfer outcome in the hormone replacement cycle

Group	LH Amplification (D1-D0)	T value	*P* value
HCG positive group	7.31 ± 3.06	1.94	0.016
HCG negative group	5.33 ± 2.84		
Clinical pregnancy group	8.01 ± 3.25	2.07	0.009
Non-clinical pregnancy group	5.04 ± 2.36		

D0 indicates the basal LH value on the second and third day of menstruation, D1 indicates the LH value on the day of endometrial transformation

## Discussion

LH is a gonadotropin secreted by the anterior pituitary gland under the stimulation of the hypothalamic gonadotropin-releasing hormone. It plays a crucial role in women for the synthesis of estrogen, ovum maturation, fertilization, and the quality of embryos [3]. LH has many other target organs in the body, which can affect the function of non-gonadal tissues. There are low levels of functional LH receptors in some non-gonadal tissues and cells [6]. These tissues and cells include gametes, early embryos or blastocysts, fallopian tubes, uterus, cervix, placenta, fetal membranes, umbilical cord, and so on [6]. Various techniques have been used to detect the receptors in these tissues and cells. The distribution of LH receptors in non-gonadal tissues is specific to certain kinds of tissues and cells, but there is no racial difference. Their role may be transmitted by protein kinase A, protein kinase C, or MAP kinase signaling pathway [7]. In vivo and in vitro experiments have revealed that the role of LH depends not only on the type of non-gonadal tissues but also on their physiological morphology. Human embryo implantation is a multistep process involving localization, adhesion, and invasion, all of which must be synchronized and functionally coordinated with embryo development and the endometrium during the “window period” of embryo implantation [4]. Other study has shown that the signal communication between the endometrium and the embryo before implantation plays a vital role in the successful implantation of the embryo [8]. The expression of various biological factors in the endometrium can be controlled by LH, one of the molecular signals involved in embryo implantation. For instance, LH inhibits the expression of insulin-like growth factor binding protein and macrophage colony-stimulating factor in the endometrium, and increases the content of leukemia inhibitory factor, vascular endothelial growth factor, and matrix metalloproteinase-9 to promote the process of endometrial remodeling and adhesion between early embryos and endometrial epithelial cells, and improve endometrial receptivity [9]. LH can also regulate the immune function of the maternal-fetal interface, reduce the rejection of endometrium by the embryo, and promote embryo implantation [10]. In the in vitro embryo implantation models, it has been observed that LH may promote embryo adhesion by up-regulating the expression levels of integrin β3 and osteopontin (OPN) on the surface of trophoblast cells [11]. The LH-R regulates endometrial function by stimulating the proliferation and maturation of endometrial cells, promoting endometrial angiogenesis, enhancing the secretion of endometrial cytokines, and modulating immune tolerance to embryos, thereby facilitating embryo implantation. The results of this study showed that women with serum LH level >11.97 U/L on the day of endometrial transformation had a higher serum HCG positive rate (*P* = 0.012) and clinical pregnancy rate (*P* = 0.022) than those with LH ≤ 11.97 U/L in the FET hormone replacement cycle cohort. Serum LH levels were 5.0–9.9 U/L in 46.27% of the women on the day of endometrial transformation. After adjustment for age, basal LH level, BMI, and AMH confounding factors, it was found that LH < 5 U/L negatively affected the HCG positive rate (OR = 0.798, 95% CI = 0.603–0.847, *P* = 0.039) and clinical pregnancy rate (OR = 0.802, 95% CI = 0.688–0.979, *P* = 0.045). Women with LH >15 U/L had a higher HCG positive rate (OR = 1.274, 95% CI = 1.034–1.699, *P* = 0.044), while those with LH >20 U/L had a higher clinical pregnancy rate (OR = 1.879, 95% CI = 1.124–3.287, *P* = 0.019).

Recent research has shown that a surge in LH has a regulatory effect on the endometrium, in addition to its essential role in inducing oocyte final maturation and ovulation. Multiple animal studies have confirmed the expression of the LH receptor in endometrial epithelial cells, glandular epithelial cells, stromal cells, decidual cells, and arterial endothelial cells, which reaches its peak during the “implantation window.” It was previously believed that LH is the primary factor inducing striking local changes during endometrial implantation [4]. After injecting high doses of HCG to induce ovulation in humans, the uterine artery resistance index decreased significantly. Based on these findings, it seems likely that LH is a crucial factor in regulating local endometrial cell maturation and functional differentiation, embryo activation, and the synchronous transformation of the endometrial receptive state. It may also facilitate the generation of uterine vascular and vascular penetration [12]. However, in the hormone replacement cycle in FET, the relationship between LH level and pregnancy has been rarely studied. The level of serum LH in the basal state and its level on the day of endometrial transformation were compared in this study. The increment of LH in the HCG positive group and clinical pregnancy group was substantially higher than that in the HCG negative group and non-clinical pregnancy group (D1 compared with D0 *P* < 0.05 in the HCG positive group; D1 compared with D0 *P* < 0.01 in clinical pregnancy group). This indicates that the LH peak in FET hormone replacement cycles is critical for discovering the optimal transfer window, and the LH peak may aid in embryo implantation.

This study has limitations as it is a retrospective study with limited cases. Moreover, we did not measure the endometrial blood flow index of the participants, which is a deficiency in this study. It should be noted that the exclusion of patients with endocrine disorders such as polycystic ovary syndrome and hypogonadotropic hypogonadism, while necessary to establish a homogeneous study population, limits the generalizability of our findings to these specific patient groups. Additionally, the notable disparity in group sizes following stratification based on LH levels represents a significant limitation that may have influenced the statistical outcomes. The influence of serum LH levels on embryo implantation in such excluded cases needs further study.

## Conclusions

Higher serum LH levels and increment on the day of endometrial transformation in the hormone replacement cycle are positively correlated with a higher clinical pregnancy rate in women with normal basal endocrine levels undergoing FET. This has encouraging implications for determining the optimal implantation window and suggests that increasing LH levels prior to frozen embryo transfer may be beneficial for embryo implantation.

## Data Availability

The datasets used and/or analysed during the current study available from the corresponding author on reasonable request.

## Abbreviations

LH: luteinizing hormone
HCG: human chorionic gonadotropin
FET: frozen-thawed embryo transfer
IVF-ET)=: in vitro fertilization-embryo transfer
BMI: body mass index
AMH: body mass index
AMH: anti-Mullerian hormone
FSH: follicle-stimulating hormone
E2: estradiol
IVF: in vitro fertilization
ICSI: intracytoplasmic sperm injection
OPN: osteopontin
PCOS: Polycystic Ovary Syndrome
HH: hypogonadotropic hypogonadism

## References

[1] Mumusoglu S, Polat M, Ozbek IY, et al. Preparation of the endometrium for frozen embryo transfer: a systematic review. Front Endocrinol (Lausanne). 2021;12:688237. 10.3389/fendo.2021.688237. 34305815 10.3389/fendo.2021.688237 PMC8299049

[2] Sekhon L, Feuerstein J, Pan S, et al. Endometrial preparation before the transfer of single, vitrified‑warmed, euploid blastocysts: does the duration of estradiol treatment influence clinical outcome? Fertil Steril. 2019;111(6):1177-1185.e3. 10.1016/j.fertnstert.2019.02.024. 31029432 10.1016/j.fertnstert.2019.02.024

[3] Liu X, Ma D, Wang W, et al. Intrauterine administration of human chorionic gonadotropin improves the live birth rates of patients with repeated implantation failure in frozen-thawed blastocyst transfer cycles by increasing the percentage of peripheral regulatory T cells. Arch Gynecol Obstet. 2019;299:1165–72. 30659362 10.1007/s00404-019-05047-6

[4] Paule SG, Heng S, Samarajeewa N, et al. Podocalyxin is a key negative regulator of human endometrial epithelial receptivity for embryo implantation. Hum Reprod. 2021;36:1353–66. 33822049 10.1093/humrep/deab032 PMC9432137

[5] Shi Y, Liu YF, Wang JM, et al. *Rhizoma drynariae* improves endometrial receptivity in a *Mus* model of dysfunctional embryo implantation. World J Tradit Chin Med. 2023;9:94–100.

[6] Lu JQ, Zhang HY. Research progress of LH/CG receptor. Reproduction and contraception. 1997;01:56–7.

[7] Fujiwara H, Araki Y, Imakawa K, et al. <article-title update="added">Dual positive regulation of embryo implantation by endocrine and immune systems – step‐by-step maternal recognition of the developing embryo. Am J Reprod Immunol. 2016;75:281–9. 26755274 10.1111/aji.12478

[8] Chu B, Zhong L, Dou S, et al. *Mirna*-181 regulates embryo implantation in mice through targeting leukemia inhibitory factor. J Mol Cell Biol. 2015;7:12–22. 25635127 10.1093/jmcb/mjv006

[9] Schumacher A, Zenclussen AC. Human chorionic gonadotropinmediated immune responses that facilitate embryo implantation and placentation. Front Immunol. 2019;10:2896. 31921157 10.3389/fimmu.2019.02896 PMC6914810

[10] Shiotani M, Matsumoto Y, Okamoto E, et al. Is human chorionic gonadotropin supplementation beneficial for frozen thawed embryo transfer in estrogen/progesterone replacement cycles?A randomized clinical trial[J]. Reprod Med Biol. 2017;16:166–9. 29259465 10.1002/rmb2.12023 PMC5661815

[11] Celik C, Asoglu MR, Karakis LS, et al. The impact of serum oestradiol concentration prior to progesterone administration on live birth rate in single vitrified–warmed blastocyst transfer cycles[J]. Reprod Biomed Online. 2019;39(6):1026–33. 10.1016/j. rbmo.2019.08.009. 31672440 10.1016/j.rbmo.2019.08.009

[12] Mumusoglu S, Polat M, Ozbek IY, et al. Preparation of the endometrium for frozen embryo transfer: a systematic review. Front Endocrinol (Lausanne). 2021;12:688237. 10.3389/fendo.2021.688237. 34305815 10.3389/fendo.2021.688237 PMC8299049

